# Lipid suppression via double inversion recovery with symmetric frequency sweep for robust 2D‐GRAPPA‐accelerated MRSI of the brain at 7 T

**DOI:** 10.1002/nbm.3386

**Published:** 2015-09-15

**Authors:** Gilbert Hangel, Bernhard Strasser, Michal Považan, Stephan Gruber, Marek Chmelík, Martin Gajdošík, Siegfried Trattnig, Wolfgang Bogner

**Affiliations:** ^1^ MR Centre of Excellence (MRCE), Department of Biomedical Imaging and Image‐guided TherapyMedical University of ViennaViennaAustria; ^2^ Christian Doppler Laboratory for Clinical Molecular MR ImagingMedical University of ViennaViennaAustria

**Keywords:** MRSI, brain MRS, parallel imaging acceleration, ultra high field, 7 T, lipid suppression, double inversion recovery, ultra‐short echo time

## Abstract

This work presents a new approach for high‐resolution MRSI of the brain at 7 T in clinically feasible measurement times. Two major problems of MRSI are the long scan times for large matrix sizes and the possible spectral contamination by the transcranial lipid signal. We propose a combination of free induction decay (FID)‐MRSI with a short acquisition delay and acceleration via in‐plane two‐dimensional generalised autocalibrating partially parallel acquisition (2D‐GRAPPA) with adiabatic double inversion recovery (IR)‐based lipid suppression to allow robust high‐resolution MRSI. We performed Bloch simulations to evaluate the magnetisation pathways of lipids and metabolites, and compared the results with phantom measurements. Acceleration factors in the range 2–25 were tested in a phantom. Five volunteers were scanned to verify the value of our MRSI method *in vivo*. GRAPPA artefacts that cause fold‐in of transcranial lipids were suppressed via double IR, with a non‐selective symmetric frequency sweep. The use of long, low‐power inversion pulses (100 ms) reduced specific absorption rate requirements. The symmetric frequency sweep over both pulses provided good lipid suppression (>90%), in addition to a reduced loss in metabolite signal‐to‐noise ratio (SNR), compared with conventional IR suppression (52–70%). The metabolic mapping over the whole brain slice was not limited to a rectangular region of interest. 2D‐GRAPPA provided acceleration up to a factor of nine for *in vivo* FID‐MRSI without a substantial increase in *g*‐factors (<1.1). A 64 × 64 matrix can be acquired with a common repetition time of ~1.3 s in only 8 min without lipid artefacts caused by acceleration. Overall, we present a fast and robust MRSI method, using combined double IR fat suppression and 2D‐GRAPPA acceleration, which may be used in (pre)clinical studies of the brain at 7 T. © 2015 The Authors. NMR in Biomedicine published by John Wiley & Sons Ltd.

**Abbreviations used**
1D/2D/3D: one‐/two‐/three‐dimensional
ACS: autocalibration signal
AD: acquisition delay
AP: anterior–posterior
BET: brain extraction tool
CAIPIRINHA: controlled aliasing in parallel imaging results in higher acceleration
Cho: choline
Cr: creatine
CRLB: Cramér–Rao lower bound
CSDE: chemical shift displacement error
DIR: double inversion recovery
EPSI: echo planar spectroscopic imaging
FID: free induction decay
FIDLOVS: FID acquisition, localised by outer volume suppression
FOV: field of view
FWHM: full width at half‐maximum
g‐factor: relative noise enhancement factor
Glx: glutamate + glutamine
GRAPPA: generalised autocalibrating partially parallel acquisition
GRE: gradient echo
GUI: graphical user interface
IQR: interquartile range
IR: inversion recovery
LR: left–right
MM: macromolecule
MP2RAGE: magnetisation‐prepared 2 rapid acquisition gradient echoes
MUSICAL: multichannel spectroscopic data combined by matching image calibration data
NAA: N‐acetylaspartate
NIR: no inversion recovery
OVS: outer volume suppression
PEPSI: proton EPSI
PI: parallel imaging
PRESS: point‐resolved spectroscopy
R: acceleration factor
R_eff_: effective acceleration factor
ROI: region of interest
SAR: specific absorption rate
SD: standard deviation
semi-LASER: semi‐localized adiabatic spin‐echo refocusing
SENSE: sensitivity encoding
SIR: single inversion recovery
SNR: signal‐to‐noise ratio
SWAMP: sequence for water suppression with adiabatic modulated pulses
tCho: total choline
tCr: total creatine
TI_1_: first inversion time
TI_2_: second inversion time
tNAA: total N‐acetylaspartate
UHF: ultrahigh field
VD: variable density
WURST: wideband, uniform rate and smooth truncation

## Introduction

Proton MRSI is an important technique with which to non‐invasively investigate the spatial distribution of various important brain metabolites. Changes in metabolite concentrations can offer insights into brain tumours, epilepsy, multiple sclerosis and other neurodegenerative diseases 1, 2, 3, 4. At ultrahigh fields (UHFs), such as 7 T, MRSI benefits from increased signal‐to‐noise ratios (SNRs), as well as the possibility to quantify more metabolites as a result of a better separation of neighbouring resonances 5. The SNR gain, in particular, could be translated into either higher spatial resolution or faster scanning.

However, there are several technical limitations for UHF MRSI: stricter specific absorption rate (SAR) constraints; SNR loss as a result of shorter *T*_2_ relaxation times; spatially variable flip angles caused by *B*_1_^+^ inhomogeneities; less reliable lipid/water suppression; increased chemical shift displacement errors (CSDEs); and larger *B*_0_ inhomogeneities 6, 7.

Several approaches have been proposed to address these problems. Sequences using the direct, echo‐less acquisition of the free induction decay (FID) signal 5, 8, 9 circumvent the SNR loss as a result of shorter *T*_2_ times and *J*‐coupling modulation. CSDEs and sensitivity to *B*_1_^+^ inhomogeneities can be reduced via the use of adiabatic refocusing pulses 10, 11, 12, adiabatic localisation pulses 13 or the omission of selective refocusing pulses 8, 14, 15. *B*_1_^+^ inhomogeneities can be reduced by improved coil hardware, such as the use of multichannel transmit coils 14. Higher order shim systems 16 and dynamic shimming 17, 18 can reduce *B*_0_ inhomogeneities.

Although there have been many reports on technical innovations for MRSI at 7 T, only a few have been able to translate the increased available SNR into higher spatial resolution. Traditional phase‐encoding schemes prohibit the acquisition of large matrix sizes in clinically feasible scan times, and faster acquisition of high‐resolution MRSI data via simultaneous spectral–spatial sampling [e.g. spiral, echo planar spectroscopic imaging (EPSI)] 19, 20, 21, 22, 23 remains challenging as a result of substantially increased gradient hardware requirements 24. However, acceleration via parallel imaging (PI) 25, 26 has been shown to be practical in reducing total measurement times. To date, only sensitivity encoding (SENSE) MRSI 27 has been used at 7 T 25, 26 with effective acceleration factors (*R*) in the range 2–9. Higher possible maximum acceleration can be expected at higher field strengths 28.

Unfortunately, PI reconstruction cannot always fully unfold the aliasing of cranial lipids that can severely compromise the spectral quality inside the brain, especially if lipid suppression is not effective 29. In particular, the quantification of *N*‐acetylaspartate (NAA) can be biased when global *B*_0_ homogeneity is insufficient 30.

Several groups have proposed improved lipid suppression schemes for 7 T 8, 14, 25, 31, as well as large matrix sizes and Hamming filtering to reduce lipid and macromolecule (MM) signal spread 5, or the use of an additional coil to crush unwanted lipid signal 32. Often, robust lipid suppression schemes at 7 T have either large power prerequisites that require a long TR, which diminishes the gain by acceleration 25, 26, or require extra hardware that may not always be available 14, 32.

In particular, when large brain coverage is necessary [i.e. three‐dimensional (3D), multi‐slice], many of these lipid suppression methods face severe limitations in the presence of strong *B*_1_^+^ and *B*_0_ inhomogeneities and in the investigations of cortical regions. Therefore, inversion recovery (IR)‐based lipid suppression techniques for full brain MRSI 33, 34 have already been proposed at 3 T. These methods utilise the different longitudinal relaxation times of brain metabolites and lipids. At the time of the excitation pulse, lipid magnetisation undergoes a zero passage and is therefore nulled, whereas metabolite magnetisation is non‐zero, but reduced.

To reduce the long measurement times of high‐resolution MRSI, whilst maintaining lipid contamination at acceptable levels, we propose a combination of FID‐MRSI with a short acquisition delay (AD) 5 and acceleration via in‐plane two‐dimensional generalised autocalibrating partially parallel acquisition (2D‐GRAPPA) 35, 36 and IR‐based lipid suppression. The improved adiabatic IR‐based lipid and MM suppression accounts for possible GRAPPA artefacts and allows robust high‐resolution MRSI of the brain at 7 T in clinically feasible scan times.

## Experimental Details

### Subjects and hardware

Five healthy volunteers (three men, two women; age 28 ± 2 years) participated in this study. Institutional Review Board approval and written informed consent were obtained. The study was performed on a 7 T whole‐body MR scanner (Magnetom, Siemens Healthcare, Erlangen, Germany) with IDEA VB17 and a 7T_SC72CD gradient system, with a total gradient strength of 70 mT/m and a nominal slew rate of 200 mT/m/s. We used a head coil with a 32‐channel receive coil array combined with a volume coil for transmission (Nova Medical, Wilmington, MA, USA).

### Sequence design

The application of in‐plane 2D‐GRAPPA 35, 36 acceleration to a high‐resolution FID‐MRSI sequence 5 reduced the measurement times at the expense of decreased metabolite SNR. The MRSI sequence had an ultrashort AD of 1.3 ms that featured high SNR as a result of negligible *T*_2_ decay and *J* modulation, and also minimised CSDEs.

**Figure 1:**
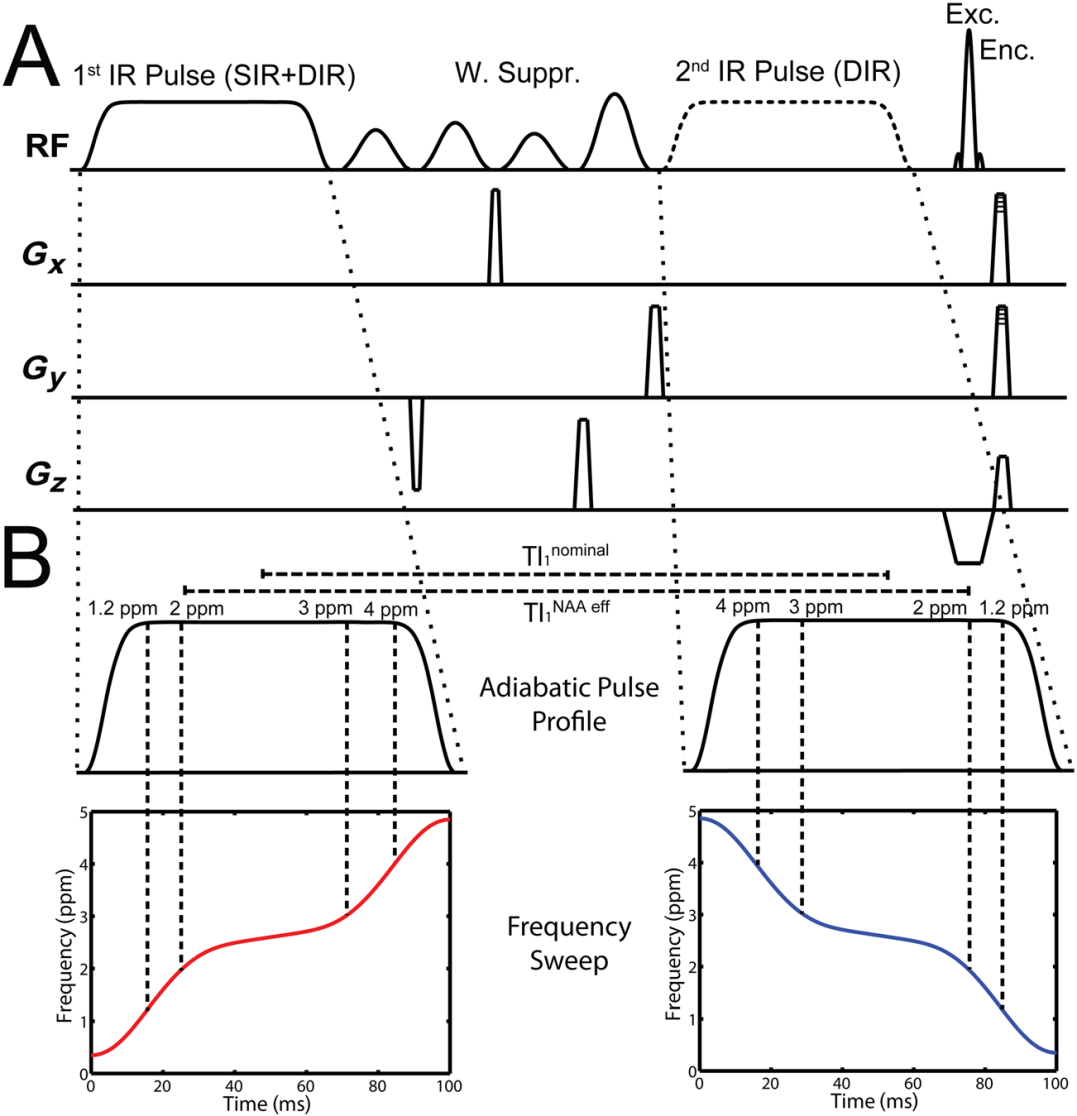
(A) Visualisation of the excitation and gradient scheme of the single inversion recovery (SIR) free induction decay (FID) sequence, using only the first inversion pulse, and the double inversion recovery (DIR) sequence, using both inversion pulses. (B) As a result of the frequency sweep over a non‐negligible pulse time, resonances are inverted at different times, as illustrated by the TI_1_ DIR of *N*‐acetylaspartate (NAA). Note that the frequency sweep of the second inversion pulse is inverted.

To achieve the suppression of artefacts caused by subcutaneous lipids (often caused by subject movement and GRAPPA aliasing), we added an improved non‐selective adiabatic IR‐based lipid and MM suppression module. The basic sequence with no inversion recovery (NIR) pulses was extended with a single inversion recovery (SIR) and a double inversion recovery (DIR) module (Fig. 1A). We defined the inversion time for SIR (TI^SIR^) as the time between the centre of the inversion pulse and the centre of the excitation pulse. For DIR, we defined $TI_{1}^{DIR}$ as the duration between the centres of both inversion pulses and $TI_{2}^{DIR}$ as identical to TI^SIR^. For non‐negligible inversion pulse durations, we had to consider that inversion does not occur simultaneously over all frequencies, as the frequency sweep is distributed over the whole pulse duration. Instead, the choice of the direction of the frequency sweep can prolong or shorten the effective TI (TI_eff_) for a particular frequency position (Fig. 1B), leading to different effective TIs for different metabolites. This is beneficial for allowing metabolites to have smaller TI_eff_ values than those for lipids, leading to higher metabolite SNRs. A major consideration for the pulse duration was the SAR limits at 7 T as a result of the quadratic dependence of SAR on *B*_1_. Increasing the pulse length to the maximum allowed by the amplifier system, 100 ms, was essential to produce a sequence applicable to *in vivo* conditions. We defined the total TR as the time between two excitation pulses, i.e. the IR times TI^SIR^ or $TI_{1}^{DIR}$ + $TI_{2}^{DIR}$ were defined to be part of TR. The base TR was defined as the TR without these IR times and was the same for the NIR, SIR and DIR sequences. A comparison between NIR, SIR and DIR was performed to determine an optimal measurement protocol for lipid suppression. Similar investigations have been performed previously 33, but under different conditions, i.e. long TRs and homogeneous excitation angles.

### Simulations and optimisations

In order to estimate the signal reduction behaviour for lipids and metabolites as a result of IR, we iteratively solved the Bloch equations using 30 iterations for the magnetisation undergoing one (SIR) or two (DIR) inversions, followed by an excitation. The simulations assumed *T*_1_ 37 and *T*_2_ 38 relaxation times for brain metabolites at 7 T, as reported previously. Lipid *T*_1_ times were determined from an *in vivo* IR experiment to be 725/405/334/333/883 ms for the 0.9/1.2/2.0/2.2/2.8 ppm lipid resonances, respectively.

We simulated the steady‐state behaviour of longitudinal and transversal magnetisation for lipids and the main metabolites NAA, creatine (Cr) and choline (Cho) over a TR range of 600–1500 ms, as well as a TI range of 100–400 ms. Optimal timings for lipid suppression considering SAR constraints were determined as described below in the ‘Measurement parameters’ section, and were validated by phantom measurements.

We calculated the optimal excitation flip angles of all major brain metabolites (NAA, Cr, Cho, glutamate, inositol) based on their *T*_1_ values and then used a geometrical average of the optimal excitation flip angles of each metabolite independently for all three sequences, as proposed by Bottomley and Ouwerkerk 39. These excitation flip angles were verified in phantom experiments and applied to *in vivo* scans. The flip angle calculations were also used to analyse the effect of *B*_1_^+^ variations on the signal intensity obtained. Further, we calculated the magnetisation pathways using the chosen measurement parameters of the NIR, DIR and SIR sequences for NAA, Cr and Cho in order to compare the simulation results (i.e. metabolite SNR, lipid suppression efficiency) with results from phantom measurements.

### Measurement parameters

The basic parameters for the NIR, SIR and DIR sequences used in phantoms and volunteers were as follows: base TR of 1038 ms, TI^SIR^ of 270 ms, $TI_{1}^{DIR}$ of 210 ms and $TI_{2}^{DIR}$ of 52 ms (the shortest timing to remain within the allowed SAR limits for DIR); four preparation scans; AD of 1.3 ms; a 64 × 64 matrix with an elliptical sampling scheme and spiral‐like *k*‐space sampling starting at the *k*‐space centre; field of view (FOV) of 220 × 220 mm^2^; slice thickness of 10 mm; a nominal resolution of 3.4 × 3.4 × 10 mm^3^; 2048 FID sampling points; and a receive bandwidth of 6000 Hz. The excitation pulse duration was 0.6 ms with optimised flip angles of 56° for NIR, 117° for SIR and 65° for DIR, as determined by our simulations and validated by phantom experiments. A four‐pulse WET water suppression 40 was used with shortened duration (i.e. 45 ms and a suppression bandwidth of 100 Hz) as optimised for our gradient system performance.

All MRSI sequences included a short (i.e. ~4 s) gradient echo (GRE)‐based prescan and acquisition of noise data. GRE prescan data were used as autocalibration signal (ACS) lines in the GRAPPA reconstruction, as well as for the coil combination 41. Receiver noise data were used for SNR calculation and noise decorrelation 42, 43 of the data obtained from individual receive coil elements.

For GRAPPA acceleration, encoding steps in *k* space for both phase‐encoding directions, i.e. anterior–posterior (AP) and left–right (LR), were omitted and accelerations between 1 and 5 in each direction were tested independently. The total nominal acceleration factor is the product of both accelerations. The *k*‐space centre was fully sampled up to the variable density (VD) radius 44. This led to *R*_eff_ being smaller than the nominal *R*. All acceleration factors are given with respect to elliptical encoding.

The inversion pulses were designed as 100‐ms‐long, 40th‐order WURST (wideband, uniform rate and smooth truncation) pulses, with a bandwidth of 1300 Hz and a delta frequency of –2.1 ppm relative to water, allowing effective inversion under consideration of the pulse profile in the range 0.9–4.3 ppm. Water suppression (minimal duration, 45 ms) was placed between the inversion pulse and excitation for SIR, and between the two inversion pulses for DIR, leading to a possible minimum TI^SIR^ of 96 ms and possible minimum $TI_{1}^{DIR}/TI_{2}^{DIR}$ of 145/52 ms. A TI^SIR^ value of 270 ms and $TI_{1}^{DIR}/TI_{2}^{DIR}$ of 210/52 ms were set. The effective TI^SIR^ values of metabolites as a result of the IR frequency sweep over 100 ms differed depending on the frequency position, e.g. 304 ms at ~1.2 ppm (lipid region), 293 ms at ~2 ppm (NAA), 250 ms at ~3 ppm (Cr) and 236 ms at ~4 ppm. For DIR, as a result of the double sweep, the effective $TI_{1}^{DIR}$ values were 278 ms at 1.2 ppm, 256 ms at 2 ppm, 170 ms at 3 ppm and 142 ms at 4 ppm, and $TI_{2}^{DIR}$ values were 18 ms at 1.2 ppm, 29 ms at 2 ppm, 72 ms at 3 ppm and 86 ms at 4 ppm (see Fig. 1). The combination of longer *T*_1_ relaxation times of metabolites and shorter effective TIs as a result of the frequency sweep maximises the metabolite signal that can be obtained, as the metabolite magnetisation has relaxed very little at the time at which the lipid magnetisation reaches zero.

### Phantom scans: GRAPPA acceleration and *g*‐factors

In order to assess the limits of possible acceleration with our hardware set‐up (i.e. 32‐channel coil at 7 T), we performed phantom measurements to test how the relative noise enhancement factors (*g*‐factors) of the MRSI sequence increase with higher acceleration. The *g*‐factor is a multiplication factor that describes the SNR loss for PI in addition to that expected as a result of the acquisition of fewer *k*‐space samples, and is defined as:

$$g-\text{factor}=\frac{SNR_{\text{notaccelerated}}}{SNR_{\text{accelerated}}\cdot \sqrt{R_{\text{eff}}}}$$

For this purpose, a fully elliptically sampled, single‐slice MRSI scan with the same parameters as the aforementioned NIR sequence, except for TR = 600 ms, was performed using a dedicated MRS phantom (Siemens, spherical, 17 cm in diameter, containing 8.2 g of NaC_2_H_3_O_3_ and 9.6 g of C_3_H_5_O_3_Li per kilogram of distilled H_2_O). The acquired data were processed fully sampled, as well as with all possible simulated GRAPPA patterns up to 5 × 5, and with VD radii in the range 1–10 for 3 × 3 acceleration. The *g*‐factors were determined based on calculated SNR values for the acetate peak (SNR calculation as described for NAA in the ‘Data processing’ section). The *g*‐factor was calculated for all in‐phantom voxels and was calculated as above.

### Phantom scans: lipid suppression and metabolite SNR

To validate the simulations for the lipid suppression efficiency, we measured fully sampled NIR, SIR and DIR sequences in an in‐house‐built spherical phantom containing brain metabolites in physiological concentrations and with a *T*_1_ value similar to that of grey matter, and a diameter of 16 cm with an added outer layer of a corn oil‐saturated textile. The fully sampled data and the data with different simulated GRAPPA patterns (2 × 2, 3 × 2, 3 × 3) were compared. The lipid signal was estimated for voxels acquired from the lipid layer using two different frequency ranges (0–2 and 0.75–1.75 ppm) by integrating the signal over the selected range. Ratios for the lipid signal integral (i.e. SIR/NIR and DIR/NIR) were compared with those predicted by simulations.

To avoid a bias in metabolite SNR determination as a result of overlay with lipid contamination, control measurements (i.e. NIR, SIR and DIR scans) were performed without the oil‐containing layer. NAA, Cr and Cho SNR values were then calculated for a circular region of interest (ROI) in the phantom centre consisting of 109 voxels. The corresponding ratios for the metabolite SNRs, i.e. SIR/NIR and DIR/NIR, were compared with the simulation results.

### Volunteer measurements

Based on the sequence settings validated in the phantoms, we defined the following session protocol for all volunteer measurements. Auto‐align, as provided by the manufacturer, was used to ensure similar slice positioning in the brain among all five volunteer scans. *T*_1_‐weighted anatomical reference images were acquired via the magnetisation‐prepared 2 rapid acquisition gradient echoes (MP2RAGE) sequence 45 with GRAPPA 4 and a measurement time of 4 min and 39 s. Additional *B*_1_^+^ and *B*_0_ maps were acquired for pulse amplitude adjustment and to ensure adequate *B*_0_ homogeneity.

After these preparation scans, the NIR, SIR and DIR scans were performed with similar scan parameters and with the same position/orientation. An *R* value of 9 using 3 × 3 GRAPPA and a VD radius of 3 resulted in measurement times of 6 min 17 s, 7 min 53 s and 7 min 51 s for NIR, SIR and DIR, respectively. The corresponding unaccelerated measurement times would have been 52 min, 65 min 30 s and 65 min, respectively, resulting in an *R*_eff_ value of 8.3. The overall measurement time excluding adjustments was 26 min and 17 s. All slices were placed transversally to cover the centrum semiovale (Fig. 8, see later).

### Data processing

We employed an in‐house‐developed software tool using Matlab (R2013a, MathWorks, Natick, MA, USA), Bash (version 4.2.25, Free Software Foundation, Boston, MA, USA) and MINC (MINC tools; v2.0; McConnell Brain Imaging Center, Montreal, QC, Canada), featuring a GUI for automatic data processing 46. As a first step, brain masks were created from the *T*_1_‐weighted images using the brain extraction tool BET2 47. The under‐sampled data were reconstructed via a 2D‐GRAPPA operator method 36 using the GRE pre‐scan data as ACS lines. After GRAPPA reconstruction, the coil combination was performed with MUSICAL 41. MUSICAL automatically performs correct zero‐order phasing during coil combination based on pre‐scan data. Additional spatial Hamming filtering was used.

The resulting spectra within the whole‐brain region were processed with LCModel software 48. We calculated SNR values for the NAA signal using an adapted pseudo‐replica method 49. The noise pre‐scan data were used to generate a 64 × 64 × 100 matrix of Gaussian noise for each coil channel, which was processed in the same way as the MRSI data. The standard deviation of this processed noise matrix and the NAA peak heights of the LCModel fits were then used to calculate SNR values.

Maps of the metabolite signal amplitudes, full width at half‐maximum (FWHM) and Cramér–Rao lower bound (CRLB) were created, together with maps of NAA SNR and frequency shift. All maps were interpolated to a 128 × 128 matrix. The mean, standard deviation and median of the datasets were calculated. For the phantom lipid signal measurements, paired *t*‐tests between DIR/NIR and SIR/NIR data were conducted.

## Results

### Simulations

**Figure 2:**
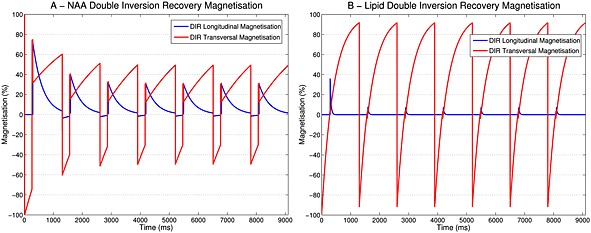
Simulated steady‐state behaviour of longitudinal and transversal magnetisation of *N*‐acetylaspartate (NAA) (A) and the 1.2‐ppm lipid resonance (B) for the double inversion recovery (DIR) sequence. The steady state was reached after four excitations. The simulations were conducted for uniform spins on‐resonance to *B*_0_. For (A), the parameters were *T*_1_ = 1860 ms, *T*_2_ = 341 ms, TI_1_^DIR^eff = 258 ms, TI_2_^DIR^eff = 52 ms, TR_base_ = 1038 ms and TE = 1.3 ms. For (B), *T*_1_ = 405 ms, *T*_2_ = 100 ms, TI_1_^DIR^eff = 278 ms, TI_2_^DIR^eff = 18 ms, TR_base_ = 1038 ms and TE = 1.3 ms. Transversal NAA magnetisation appears lower than expected in steady state but, as shown in Fig. 3, it needs to be compared with the steady‐state magnetisation of the no inversion recovery (NIR) sequence that features ~50% of maximum magnetisation.

**Figure 3:**
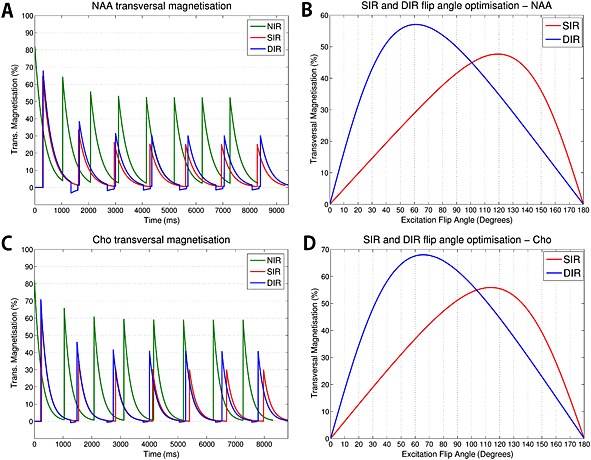
Simulated steady‐state behaviour of transversal magnetisation of *N*‐acetylaspartate (NAA) (A) and choline (Cho) (C) for no inversion recovery (NIR), single inversion recovery (SIR) and double inversion recovery (DIR), as well as the magnetisation behaviour of NAA (B) and Cho (D) relative to the excitation flip angle for SIR and DIR. The simulations were conducted for uniform spins on‐resonance to *B*_0_. Flip angles of 56°/117°/65° (averages over the most important metabolites) were used for NIR/SIR/DIR, as well as TR_base_ = 1038 ms and TE = 1.3 ms. For NAA, *T*_1_ = 1860 ms, *T*_2_ = 341 ms, TI^SIR^eff = 293 ms, TI_1_^DIR^eff = 258 ms and TI_2_^DIR^eff = 52 ms were used. For Cho, *T*_1_ = 1415 ms, *T*_2_ = 230 ms, TI^SIR^eff = 250 ms, TI_1_^DIR^eff = 170 ms and TI_2_^DIR^eff = 72 ms were used.

Figure 2 shows that the DIR steady‐state signal of both longitudinal and transversal magnetisation for NAA was reached after four excitations. The same was true for a comparison of the transversal magnetisation of NAA and Cho in NIR, SIR and DIR (Fig. 3A, C). SIR lost more magnetisation than DIR compared with NIR. The optimal average flip angles for all metabolites were 56° for NIR, 117° for SIR and 65° for DIR. Figure 3B, D shows values for NAA and Cho. The signal obtained for different excitation flip angles around the optimised angle was more stable with DIR than with SIR. For SIR, flip angle deviations of 10%/15%/20% reduced magnetisation to 97%/92%/85%, respectively, but only to 99%/98%/97% for DIR. This indicates a higher robustness of DIR to *B*_1_^+^ inhomogeneities.

Metabolite and lipid signal simulation results (Tables 1 and 2) showed, as expected, a similar lipid suppression (89% for SIR and 90% for DIR), but DIR had a 45% higher NAA signal than SIR.

**Table 1:** Lipid signals of double inversion recovery (DIR) and single inversion recovery (SIR) relative to no lipid suppression (no inversion recovery, NIR) obtained in a phantom for different acceleration factors, compared with simulation results. Lipid amplitudes were calculated by integrating the frequency range of 0–2 ppm or 0.75–1.75 ppm. Two ranges were used in order to achieve a more robust comparison

0–2‐ppm region	Mean ± SD (%)	0.75–1.75‐ppm region	Mean ± SD (%)
DIR			
Simulated	10.0	Simulated	10.0
*R* = 1	5.3 ± 1.8	*R* = 1	5.5 ± 1.4
*R* = 4	5.8 ± 2.2	*R* = 4	6.0 ± 1.6
*R* = 6	6.6 ± 4.0	*R* = 6	6.3 ± 2.6
*R* = 9	7.9 ± 5.0	*R* = 9	7.0 ± 3.0
SIR			
Simulated	11.4	Simulated	11.4
*R* = 1	15.2 ± 1.3	*R* = 1	15.6 ± 1.4
*R* = 4	15.9 ± 2.6	*R* = 4	15.7 ± 2.0
*R* = 6	15.3 ± 1.6	*R* = 6	15.6 ± 1.6
*R* = 9	16.1 ± 2.2	*R* = 9	15.9 ± 2.1

SD, standard deviation.

**Table 2:** Metabolite signal‐to‐noise ratios (SNRs) of double inversion recovery (DIR) and single inversion recovery (SIR) relative to the no inversion recovery (NIR) sequence, comparing simulation results and measurements with different acceleration factors for *N*‐acetylaspartate (NAA), creatine (Cr) and choline (Cho) in a phantom

	DIR	Mean ± SD (%)	SIR	Mean ± SD (%)
NAA	Simulated	57.0	Simulated	48.1
	*R* = 1	51.7 ± 1.9	*R* = 1	36 ± 6.4
	*R* = 2 × 2	53.3 ± 4.7	*R* = 2 × 2	36.9 ± 7.6
	*R* = 3 × 2	51 ± 10	*R* = 3 × 2	35.1 ± 8.2
	*R* = 3 × 3	52.7 ± 4.7	*R* = 3 × 3	35.5 ± 7.8
Cr	Simulated	70.8	Simulated	54.3
	*R* = 1	70 ± 12	*R* = 1	27.6 ± 6.5
	*R* = 2 × 2	69 ± 13	*R* = 2 × 2	25.8 ± 7.6
	*R* = 3 × 2	72 ± 15	*R* = 3 × 2	31 ± 13
	*R* = 3 × 3	74 ± 13	*R* = 3 × 3	29.6 ± 8.8
Cho	Simulated	72	Simulated	56.5
	*R* = 1	67.8 ± 8.2	*R* = 1	29.8 ± 3.6
	*R* = 2 × 2	66.2 ± 9.6	*R* = 2 × 2	28.4 ± 6.1
	*R* = 3 × 2	68 ± 13	*R* = 3 × 2	33 ± 11
	*R* = 3 × 3	67.7 ± 8.6	*R* = 3 × 3	31.3 ± 6.8

SD, standard deviation.

### Phantom: GRAPPA acceleration

**Figure 4:**
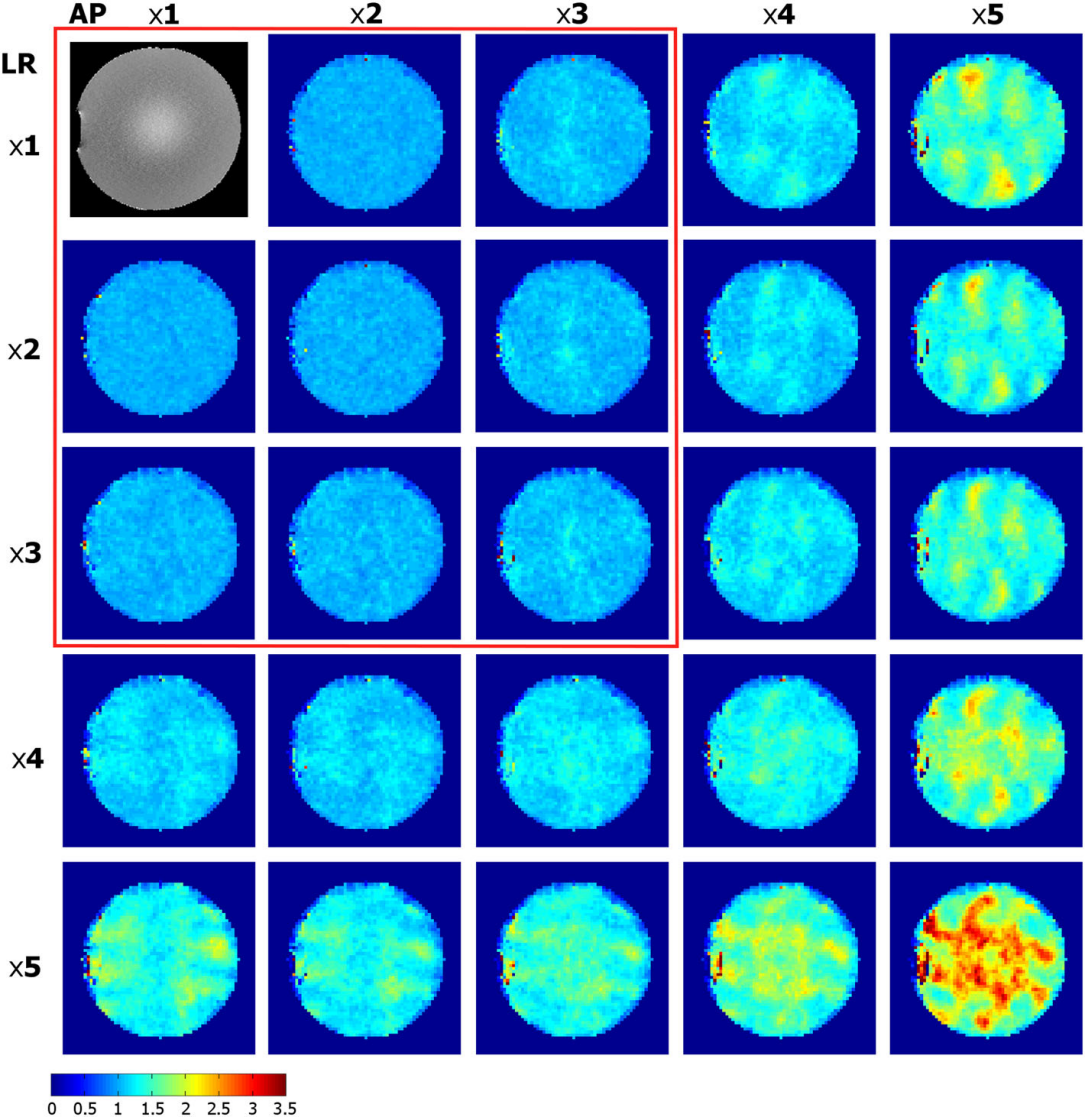
*g*‐Factor maps for generalised autocalibrating partially parallel acquisition (GRAPPA) acceleration patterns of 1–5 in the left–right (LR) and anterior–posterior (AP) directions with a variable density (VD) radius of 2 in a phantom. The red box highlights the patterns with *g*‐factors below 1.1 (Table 3) and high homogeneity.

Our GRAPPA reconstruction of MRSI data worked with a minimal loss of data quality. The median *g*‐factors were less than 1.1 for accelerations up to 3 × 3 (Fig. 4, Table 3), showing good homogeneity. Increasing the VD radius for the 3 × 3 acceleration increased the median *g*‐factors from 1.08 (VD of 1) to 1.09/1.09/1.12/1.17 (VDs of 2/3/5/10, respectively), but with interquartile ranges (IQRs) of 0.12.

**Table 3:** A comparison of the *g*‐factors [median and interquartile range (IQR)] for different acceleration patterns in the anterior–posterior (AP) and left–right (LR) directions from 1 × 1 up to 5 × 5 in a phantom, as shown in Fig. 4. Up to 3 × 3 generalised autocalibrating partially parallel acquisition (GRAPPA), the *g*‐factors remain below 1.1

Acc. L–R A–P	×1	×2	×3	×4	×5
×1	Median/IQR	1.02/0.06	1.06/0.10	1.16/0.15	1.45/0.31
×2	1.01/0.06	1.01/0.08	1.06/0.10	1.12/0.15	1.29/0.25
×3	1.04/0.09	1.04/0.09	1.08/0.12	1.17/0.16	1.37/0.29
×4	1.14/0.14	1.11/0.14	1.18/0.16	1.27/0.20	1.57/0.43
×5	1.37/0.28	1.32/0.26	1.37/0.27	1.56/0.40	2.03/0.78

### Phantom: lipid and metabolite SNR

Compared with simulations, SIR underperformed, with 84–85% lipid suppression *versus* the 89% expected from simulations, whereas DIR performed better than expected, with 90–95% suppression *versus* 90% (Table 1), i.e. a lipid suppression factor of 10–20. There was a highly significant difference between SIR and DIR suppression (*p* < 0.001).

For the metabolites, retained SNR for SIR was lower than simulated, approximately 36%/27%/30% for NAA/Cr/Cho, respectively, which was probably affected strongly by *B*_1_^+^ inhomogeneities. For DIR, the results were close to the simulated values, approximately 52%/70%/67% for NAA/Cr/Cho, respectively (Table 2).

### Volunteer measurements

A comparison of NAA SNR, FWHM and CRLBs (Table 4) for NIR, SIR and DIR showed the expected additional SNR loss as a result of the removed lipid artefacts, increased CRLBs and reduced FWHM for DIR. The average SNR values for SIR of 2–3 were already too low for reliable quantification, whereas DIR SNRs were >6.

**Table 4:** Comparison of measurement quality criteria for all five volunteers. Average signal‐to‐noise ratio (SNR), Cramér–Rao lower bound (CRLB) and full width at half‐maximum (FWHM) over the whole slice of the fitted total *N*‐acetylaspartate (tNAA) signal for no inversion recovery (NIR), double inversion recovery (DIR) and single inversion recovery (SIR) sequences. The smaller FWHM values for DIR in comparison with NIR suggest that lipids were incorrectly fitted as tNAA in the NIR measurement

tNAA	NIR			DIR			SIR		
	SNR	CRLB (%)	FWHM (Hz)	SNR	CRLB (%)	FWHM (Hz)	SNR	CRLB (%)	FWHM (Hz)
Vol. 1	21.7 ± 9.8	5 ± 5	19 ± 16	7.7 ± 3.0	9 ± 7	15 ± 13	3.0 ± 1.4	17 ± 8	20 ± 15
Vol. 2	15.8 ± 9.4	6 ± 5	22 ± 11	5.4 ± 1.9	12 ± 5	20 ± 2	2.03 ± 0.72	27 ± 10	25 ± 14
Vol. 3	20 ± 11	7 ± 5	19 ± 10	8. ± 2.9	8 ± 5	16 ± 10	3.0 ± 1.1	19 ± 9	21 ± 16
Vol. 4	16.8 ± 8.8	6 ± 5	18 ± 11	6.8 ± 2.4	9 ± 5	13 ± 8	2.46 ± 0.85	21 ± 10	22 ± 15
Vol. 5	15.4 ± 6.9	6 ± 6	20 ± 14	6.3 ± 2.8	11 ± 8	16 ± 10	2.45 ± 0.92	21 ± 9	22 ± 15

**Figure 5:**
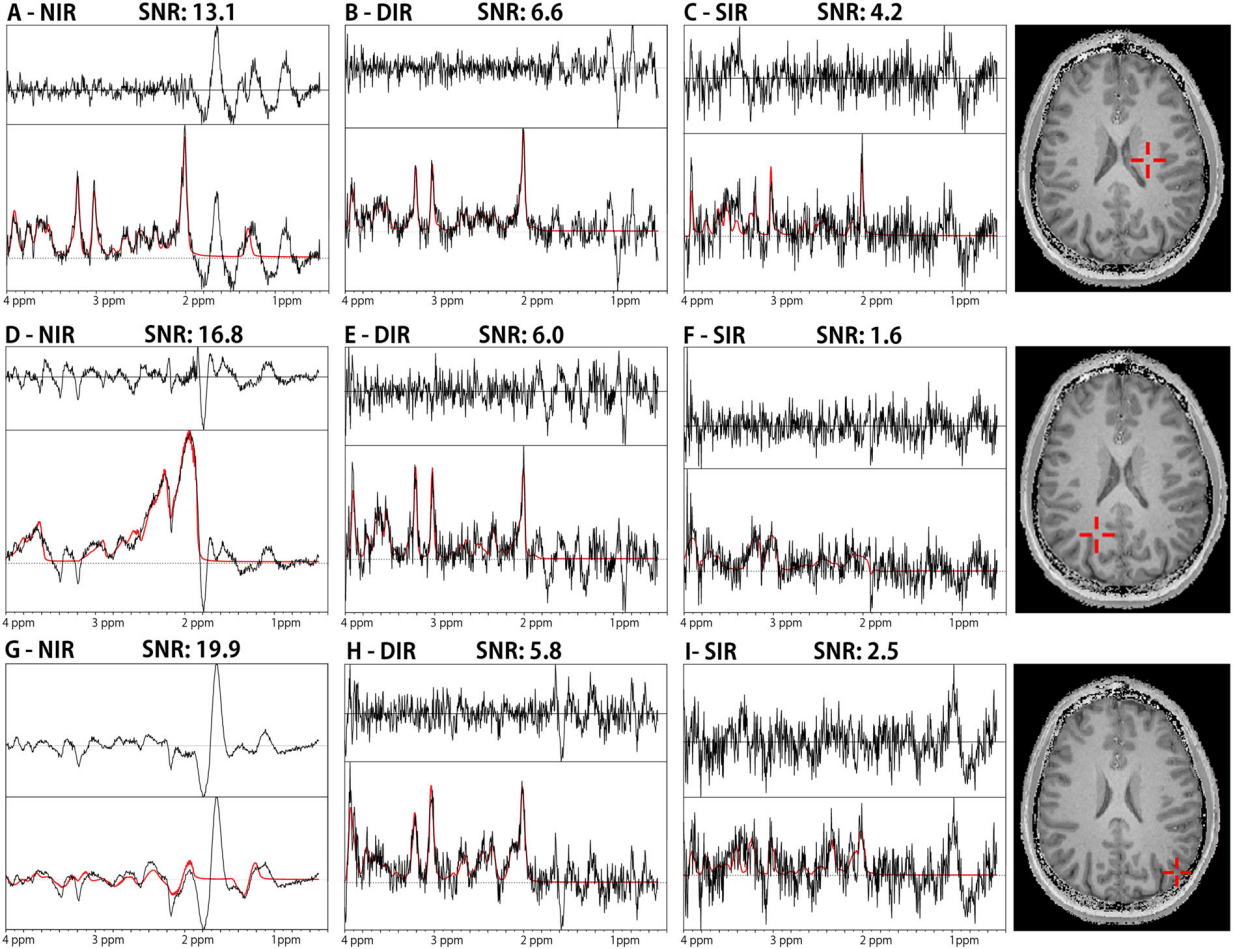
(A–C) Spectra of a central white matter voxel for no inversion recovery (NIR), double inversion recovery (DIR) and single inversion recovery (SIR). (D–F) Spectra from the position of a lipid‐fold‐in artefact. (G–I) Spectra of a grey matter voxel in proximity to the cranium in the occipital cortex. Bottom plots: measured spectra in black and LCModel fits in red. Top plots: residuum of non‐fitted signal. The spectral range of 0–4 ppm was processed. The signal‐to‐noise ratios (SNRs) stated are for the *N*‐acetylaspartate (NAA) signal of the respective voxel and method. The changed metabolite ratios of the inversion recovery (IR) methods can be accounted for in post‐processing or when evaluating the results.

**Figure 6:**
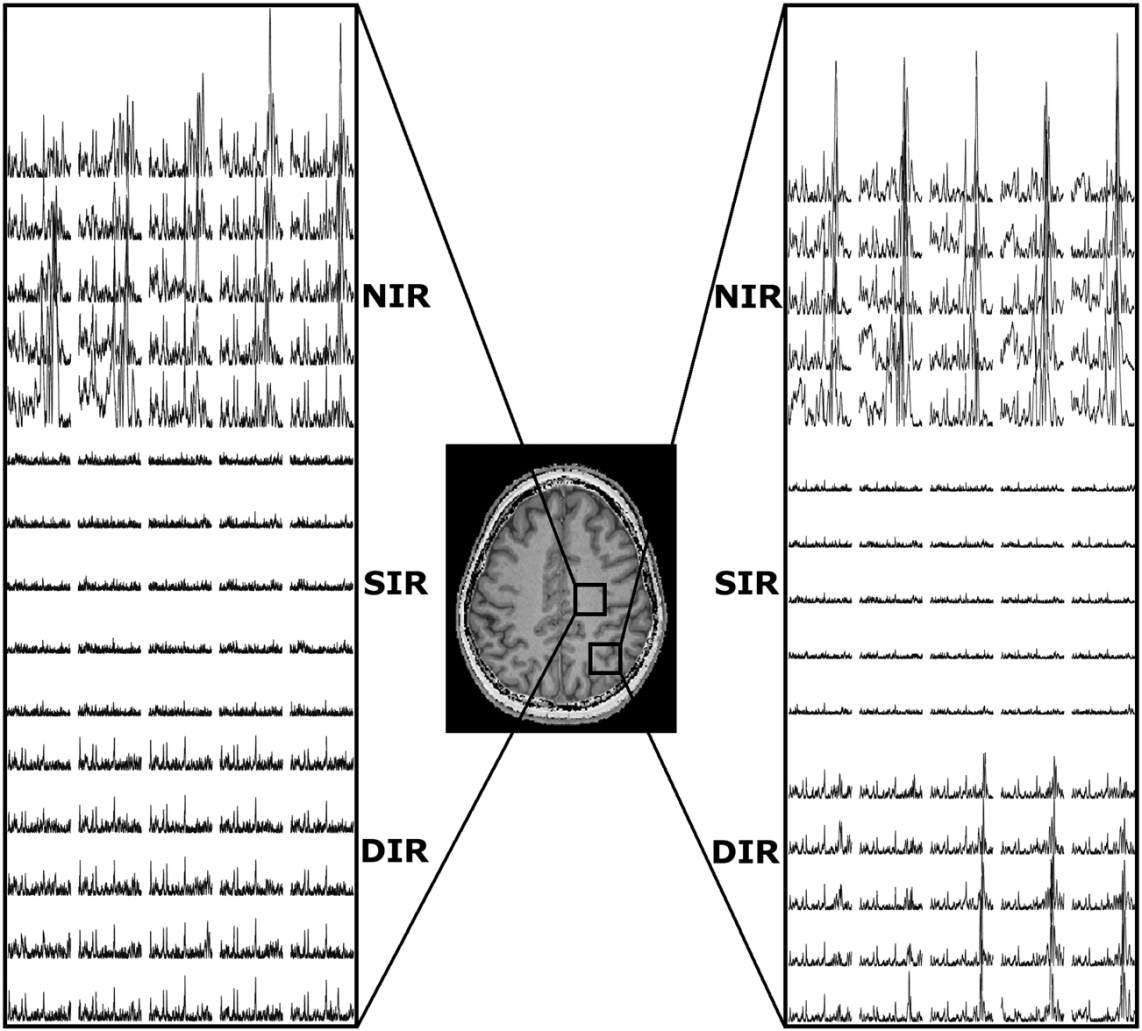
Spectral data of two 5 × 5 voxel regions for the no inversion recovery (NIR), single inversion recovery (SIR) and double inversion recovery (DIR) sequences. Left: central white matter region. Lipid signals are removed by SIR and DIR, but DIR retains more metabolite signal‐to‐noise ratio (SNR). Right: occipital region close to the cranium with a mix of grey and white matter. Lipid signals strongly overlay the *N*‐acetylaspartate (NAA) region in NIR and are removed in SIR, whereas DIR does not remove the lipid signals as efficiently around the 1‐ppm region, but is still sufficient to remove lipid contamination close to the NAA signal. Further, it retains more metabolite signal than SIR. The scale of the displayed magnitude spectra is the same in all plots.

**Figure 7:**
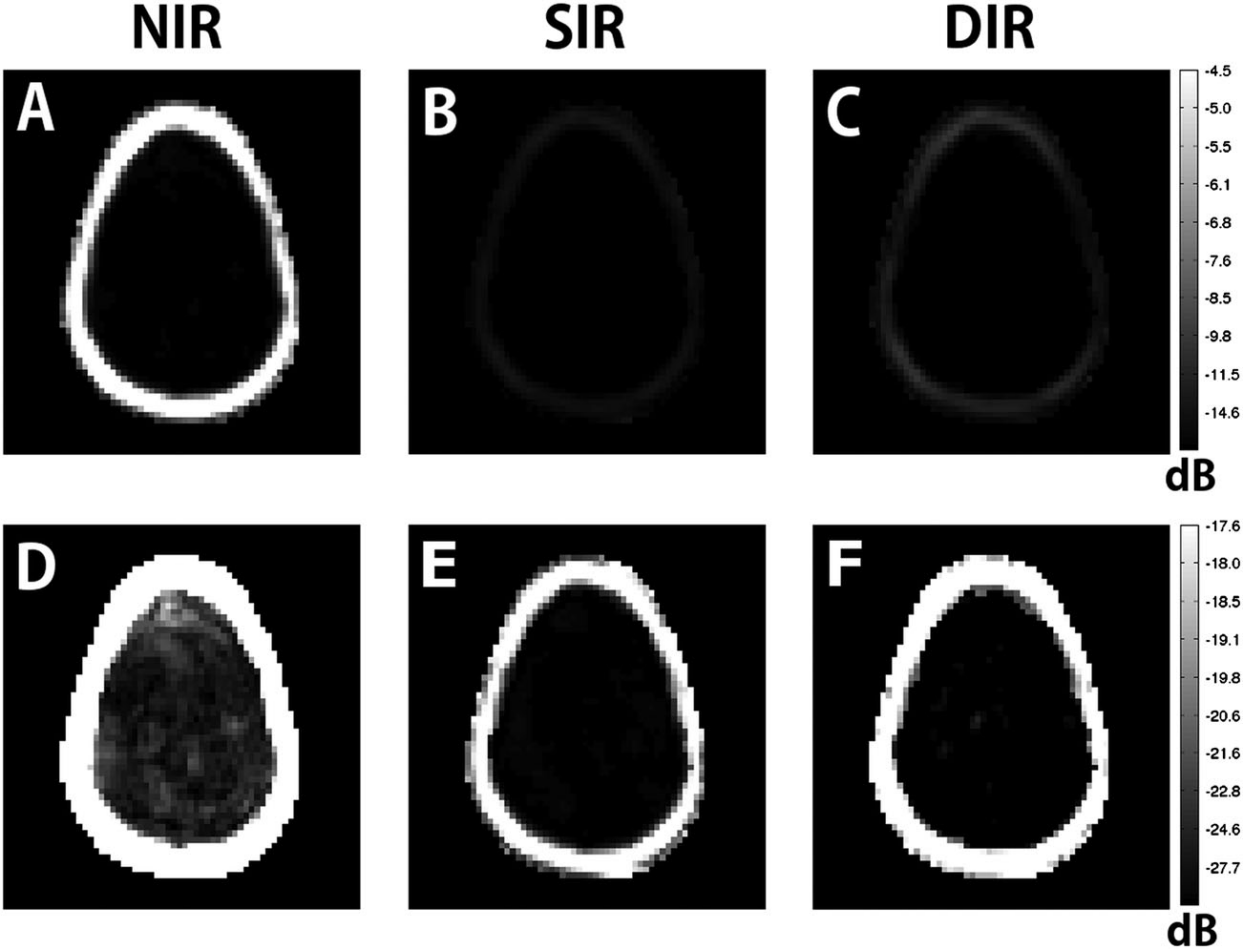
Lipid maps of volunteer 3 created by summing the 0–2‐ppm region of the spectrum of each voxel, with baseline correction for the brain voxels. No further filter beyond the elliptical *k*‐space sampling and Hamming filtering was applied. (A–C) No inversion recovery (NIR), single inversion recovery (SIR) and double inversion recovery (DIR) are scaled to half of the maximum lipid signal, whereas, in (D) and (E), they are scaled to 1/40th of the maximum lipid signal. (A–C) Suppression efficiency of SIR and DIR in comparison with NIR. (D, E) Removal of lipid artefacts inside the brain. In the volunteers, lipid suppression was slightly better for the SIR method, and DIR retained minimal lipid artefacts inside the brain.

Individual voxel spectra (Fig. 5) illustrate the removal of lipid contamination by SIR and DIR, even in the locations in which lipid artefacts overlapped strongly with metabolite signals as a result of *B*_0_ inhomogeneities. The visualisation of 5 × 5 adjacent voxels at two different locations (Fig. 6) further proves this. SIR lipid suppression seems to be slightly better than DIR at the cost of even more metabolite SNR. Evaluation of the lipid spectral region shows efficient lipid suppression over the whole slice, as illustrated by lipid signal maps (Fig. 7) and maps of the lipid region spectra (Fig. S1).

**Figure 8:**
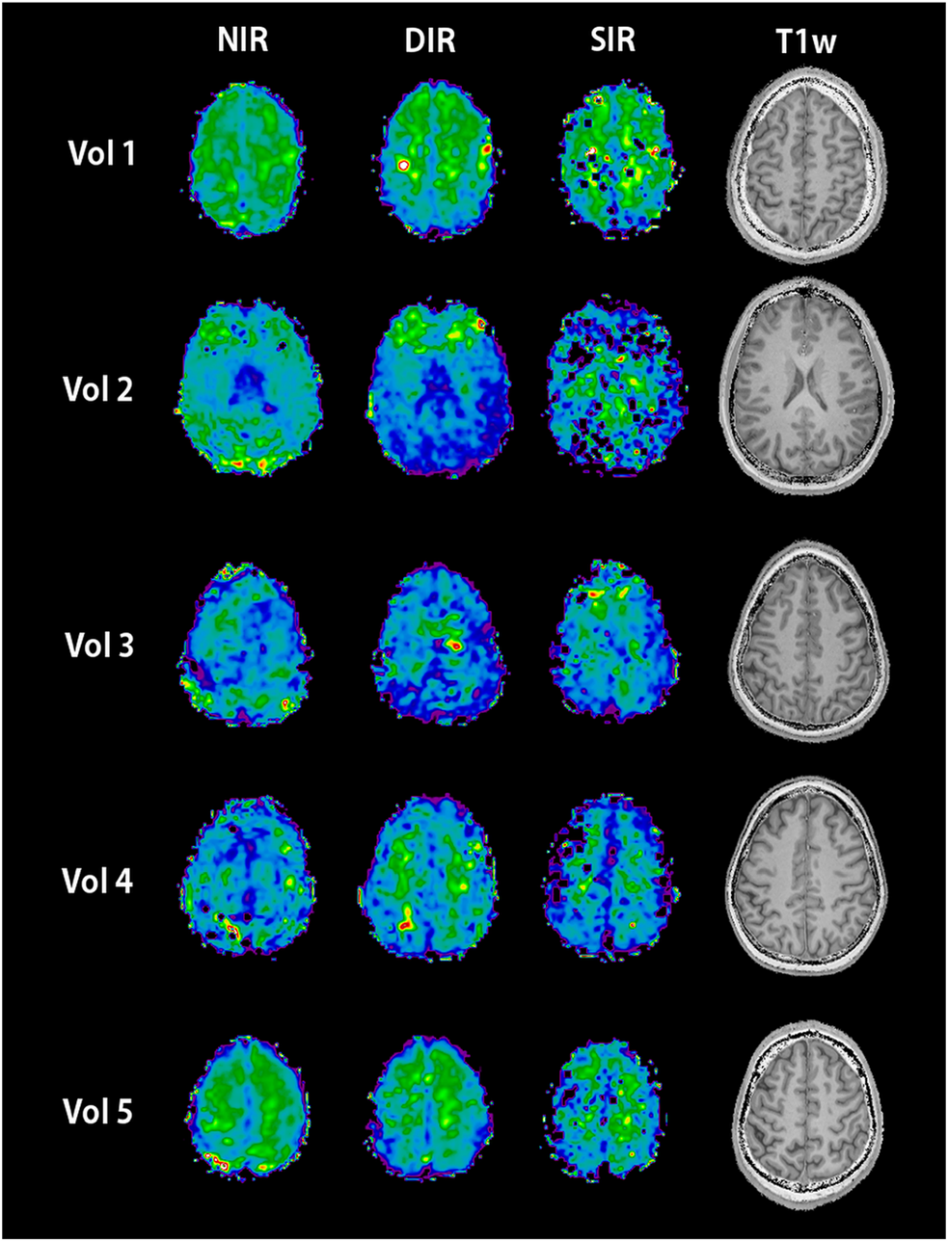
Total *N*‐acetylaspartate (NAA) maps of all five volunteers for the no inversion recovery (NIR), double inversion recovery (DIR) and single inversion recovery (SIR) sequence with an *R* value of 9, scaled relative to the individual map maxima, as well as *T*1‐weighted (T1w) images. Ring‐like parallel imaging (PI) artefacts can be clearly seen on the NIR maps.

**Figure 9:**
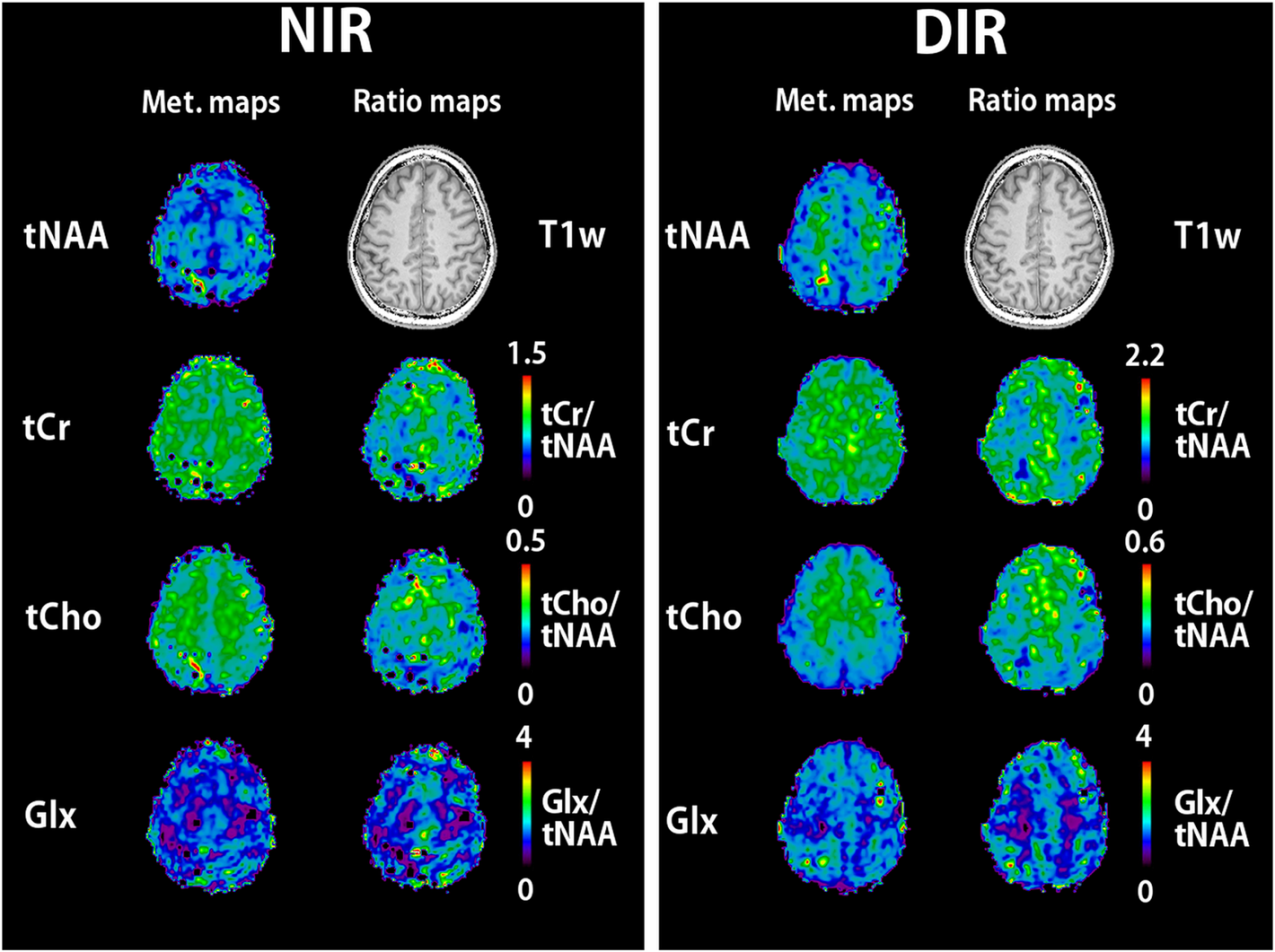
Metabolic maps of total *N*‐acetylaspartate (tNAA), total creatine (tCr), total choline (tCho) and glutamate + glutamine (Glx), as well as metabolite ratio maps (all to tNAA), for volunteer 4, measured with an *R* value of 9. Double inversion recovery (DIR) removes parallel imaging (PI) fold‐in artefacts, improving the metabolic map quality. The differences in metabolite loss as a result of DIR (e.g. more loss for NAA than for Cr and Cho) change the scaling of the metabolite ratio maps from no inversion recovery (NIR) to DIR. T1w, *T*_1_‐weighted.

NAA maps of all volunteers (Fig. 8) show the prevalence of fold‐in artefacts in the NIR maps and the stability of DIR lipid suppression whilst retaining sufficient NAA signal. For SIR, lipid suppression was also efficient, but the NAA signal was low. A more detailed examination of the metabolic and ratio maps (Fig. 9) provided further verification of the artefact removal for total *N*‐acetylaspartate (tNAA), total creatine (tCr), total choline (tCho) and glutamate + glutamine (Glx) maps. Results are in accordance with previous publications, such as Emir *et al*. 50. As a result of the different SNR loss rates for different metabolites, ratio maps require a frequency‐dependent correction of metabolite signal amplitudes.

## Discussion

In this study, we have demonstrated the use of a robust, accelerated, high‐resolution MRSI sequence with improved IR‐based lipid suppression for application in metabolic studies of the brain at 7 T.

The use of high spatial resolution at 7 T has led to a substantial increase in scan times reported in recent MRSI studies of the brain 5, 8. To provide clinically feasible scan times for future studies, these long scan times must be reduced.

At lower field strengths, long scan times have been overcome efficiently via advanced trajectories, such as EPSI or spiral MRSI 19, 21, which offer very fast sampling. However, at 7 T, the use of EPSI or spiral MRSI may be problematic without powerful gradient systems [e.g. 80 mT/m and 600 mT/m/ms as used in ref. 23 to measure a 32 × 32 matrix in 8 min and 32 s with a spectral bandwidth of 1380 Hz], because these techniques already operate at the limits of gradient performance at 3 T 24. To account for the 2.3‐fold larger necessary receiver bandwidth at 7 T, a 2.3‐fold shorter spiral/EPSI trajectory would be necessary. This would severely limit the maximum achievable matrix sizes. Overcoming these limitations may result in superior results compared with phase‐encoded MRSI.

PI, however, is known to perform better at higher field strengths 51, and the steady increase in receive coil elements for conventional array coils up to 64 has dramatically improved the performance of PI methods.

The first promising applications for PI‐accelerated MRSI were shown at 7 T by Zhu *et al*. 25 and Kirchner *et al*. 26. Both sets of authors used an *R* value of 4 and simulated accelerations up to 9. The need for high SAR outer volume suppression (OVS) schemes required a TR of 4.5 s for Zhu *et al*. 25, leading to a matrix size of 29 × 27 with a FOV of 21 × 19 and a measurement time of 12.5 min. Kirchner's FIDLOVS (FID acquisition, localised by OVS) scheme required a TR of 8 s with a 20 × 16 matrix and an unaccelerated measurement time of 42 min. For both approaches, the long TRs as a result of lipid suppression reduced the possible spatial resolution.

These studies used SENSE and were limited by SAR constraints. To our knowledge, we are the first group to use a GRAPPA‐based acceleration approach for MRSI at 7 T. Using GRE images as ACS data, we obtained high reconstruction qualities and low *g*‐factors, even for accelerations as high as *R*_eff_ = 8.3, as there was no need to obtain ACS from lengthy additional MRSI excitations. One‐dimensional (1D)‐GRAPPA has been shown previously, but only in combination with EPSI by Sabati *et al*. 21, and proton EPSI (PEPSI) by Tsai *et al*. 52, and at 3 T. In the first publication, acceleration factors higher than 4 limited the metabolite quantification, whereas the second achieved an *R*_eff_ of only 1.6 as a result of the ACS data acquired within the MRSI scan itself. 2D‐GRAPPA MRSI was proposed by Banerjee *et al*. 53, but acquiring ACS data again within the MRSI sequence reduced the acceleration from 4 to *R*_eff_ < 2.

MRSI scans that are accelerated via PI benefit from a method of lipid suppression that is sufficiently robust for routine measurements. As soon as the acquired ACS lines or SENSE maps become inaccurate, the unfolding of lipid artefacts may become imperfect, leading to possible spectral contamination. However, most suppression schemes have significantly increased TRs up to 8 s. Our accelerated MRSI sequence uses a shorter TR of 1.3 s because of our IR‐based suppression scheme with long inversion pulses.

We have compared SIR‐ and DIR‐based lipid suppression approaches. Both suppression methods eliminated lipid signals sufficiently, but DIR retained higher metabolite SNR than SIR at the cost of higher SAR. As a result of lower SNR, SIR did not allow us to perform reliable metabolite quantification. Further, the smaller DIR excitation flip angles relative to SIR enable the use of shorter excitation pulses. Our DIR suppression scheme is an improvement on previously proposed IR suppression methods, as it leads to a higher SNR and slightly higher lipid suppression performance. According to our phantom measurements, our DIR sequence reaches suppression factors of 10–20 for extracranial lipids, whereas SIR has factors of 6–7. In comparison, Ebel *et al*. 33 used SIR and DIR non‐selective suppression at 1.5 T *in vivo*, and reached DIR/SIR lipid signal ratios of 53% for TE = 70 ms and 72% for TE = 135 ms. As a result of different inversion timings, their metabolite SNR ratios of DIR/SIR were around 60%. In summary, we obtained similar lipid suppression behaviour, but longer TI^DIR^ values, and different field strengths led to a different behaviour for metabolite SNR loss, reducing SIR SNR more than DIR SNR.

With 3 T systems, several approaches to lipid suppression for MRSI acquisition schemes exist. Gu and Spielman 54 achieved *B*_1_‐insensitive water and lipid suppression using dual‐band, frequency‐selective preparation pulses with a lipid suppression factor of >100. Tsai *et al*. 52 applied OVS for GRAPPA‐PEPSI with a 32 × 32 matrix measured in 32 s for an *R* value of unity. Considering voxel volume, *R* and *B*_0_, this approach led to approximately the same SNR value as our sequence, despite the inherent SNR loss as a result of DIR. SIR 33 for GRAPPA‐EPSI has been shown recently by Sabati *et al*. 21 and Ding *et al*. 55, featuring the acquisition of a 50 × 50 × 18 matrix in 16 min, with a nominal voxel size of 0.31 cm^3^. This approach allows robust mapping of the most important metabolites, providing approximately the same NAA SNR as our method, when taking into account the voxel size, *R* and *B*_0_.

For 7 T systems, several groups have published promising lipid suppression techniques without metabolite signal loss. Boer *et al*. 9 used SWAMP and cost function‐optimised *B*_0_ shimming for FID MRSI, measuring a 32×32 matrix in 28 min. They reported a lipid suppression factor of 4–5, but were limited to the quantification of an ROI. Henning *et al*. 8 used FIDLOVS for a suppression factor of 25, at the cost of a TR of 5 s, and the measurement of a 32 × 32 matrix took 64 min. Boer *et al*. 17 have shown the use of dynamic shimming in multi‐slice MRSI combined with slice‐selective hyperbolic secant pulses for a lipid suppression factor of ~30. They measured a 20 × 20 × 5 matrix in 18 min. The dynamic shimming itself required more than 10 min of measurement time. The approach of Zhu *et al*. 25 of SENSE MRSI with dual‐band water/lipid suppression using adiabatic full passage pulses had a simulated lipid suppression factor of over 20. For a voxel volume of 0.64 cm^3^ and 2 × 2 acceleration, an SNR of ~150 was reached, which is very similar to our results if the 6.4‐fold smaller voxel volume, higher *R* and losses to IR are taken into consideration. Balchandani *et al*. 31 used a spatial–spectral adiabatic pulse and point‐resolved spectroscopy (PRESS) to measure a 12 × 12 matrix with a 9 × 7 ROI, and reported a lipid suppression factor of 7 and a loss of metabolite signal between 3.2 and 4.7 ppm.

Highly efficient lipid suppression was demonstrated by Hetherington *et al*. 14 using an eight‐channel transmission array for ring‐shaped *B*_1_^+^ excitation to suppress subcutaneous lipids, and achieved a nine‐fold suppression for SIR and a 58‐fold suppression for DIR, for the measurement of a 32 × 32 matrix in 25 min. Boer *et al*. 32 proposed the use of an additional crusher coil fitted into the head coil, which was activated for 1–2 ms during TE and reached 20–70‐fold lipid suppression, thereby negating the need for additional suppression pulses. This was coupled with ultrashort water suppression for a TR of 0.11 s, allowing the measurement of very high matrix sizes with a 3 × 3 × 10 mm^3^ resolution in 5 min, with the downside of the lack of water suppression stability over the whole slice. Both approaches work very well, but require the use of non‐standard hardware.

In contrast with other 7 T MRSI techniques that use localisation schemes, such as semi‐LASER 10 or PRESS 12, we were not limited to an ROI and obtained spectra for the whole slice and a 64 × 64 resolution. Overall, our DIR method reached a lipid suppression performance similar to that of the other non‐hardware‐augmented methods, but was not limited by long TRs, reduced ROIs or low resolutions, thus allowing the high‐resolution coverage of a whole brain slice.

### Limitations

At 7 T, a limiting factor for non‐selective adiabatic inversion pulses is SAR. To maintain adiabatic inversion with our 100‐ms‐long inversion pulses, our DIR MRSI sequence could not be run with a total TR below 1.3 s. Based on our simulations, a shorter TI/TR should lead to better lipid suppression and increased metabolite signal. However, TRs of approximately 1.5 s are commonly used in MRSI, and further inversion pulse optimisation (e.g. longer duration, improved pulse shape, tailored frequency sweep and bandwidth) could reduce SAR requirements and offer more robust lipid suppression. Further, our simulations did not account for the relaxation in the rotating frame of reference, *T*_1ρ_, during the long inversion pulse rotation 56, which may explain the differences between our simulations and the measurement results. The frequency‐dependent metabolite SNR loss alters the metabolite ratios, but can be corrected retrospectively. NAA SNR is reduced the most but, as NAA is the most prominent brain metabolite signal of interest, this is acceptable.

High acceleration factors, such as an *R* value of 9, allow for high spatial resolution in approximately 7–8 min, but may be applicable only when IR‐based metabolite signal losses are not too strong. The MUSICAL method provides an optimal coil combination, but pulse optimisation, advanced multi‐slice encoding [e.g. Hadamard encoding 57] and PI methods [e.g. CAIPIRINHA 58], as well as improved coil hardware, can further increase SNR. This will improve the reliability of the sequence and offers the possibility to use even higher acceleration factors.

As a result of the limitations of current shim hardware (i.e. second‐order *B*_0_ shimming), we were limited to acquisitions at the level of the ventricles and above. Advanced shim hardware 4, 14, 59 would allow the coverage of larger parts of the brain.

Even with the current limitations, this sequence allows the clinical investigation of metabolic changes caused by tumours, multiple sclerosis and other brain diseases. With non‐selective suppression, this method is insensitive to moderate *B*_0_ and *B*_1_^+^ inhomogeneities and could be an efficient fat suppression method for whole‐brain 3D‐MRSI, where most other suppression approaches would be difficult to perform 33.

## Conclusions

The use of nine‐fold GRAPPA acceleration for MRSI worked well, with negligible *g*‐factor penalties. Using non‐selective symmetric frequency sweep DIR, we were able to suppress lipid artefacts caused by PI fold‐in. We retained more relative metabolite SNR than previous DIR methods as a result of the shorter inversion times for the metabolite resonances, without the need for complex measurement protocols. This suppression method requires no additional hardware and provides whole‐slice metabolic mapping, even including cortical regions. One 64 × 64 slice can be acquired in approximately 8 min to quantify the prominent brain metabolites, although a reduction in *R* is necessary if greater SNR is required, especially for the observation of Glx and inositol (Ins). Overall, we present a fast and robust method for MRSI using DIR and 2D‐GRAPPA acceleration which may be used in (pre)clinical studies of metabolic deviations of the brain at 7 T.

## Supporting information

Supporting info item

Supporting info item
