# Supplementary material for: Lipid suppression via double inversion recovery with symmetric frequency sweep for robust 2D‐GRAPPA‐accelerated MRSI of the brain at 7 T
Source: NMR Biomed. 2015 Sep 15;28(11):1413–25. doi: 10.1002/nbm.3386 (PMC4973691; doi:10.1002/nbm.3386)
Supplement: Supplementary file 1 — Supporting info item [file NBM-28-1413-s001.docx]

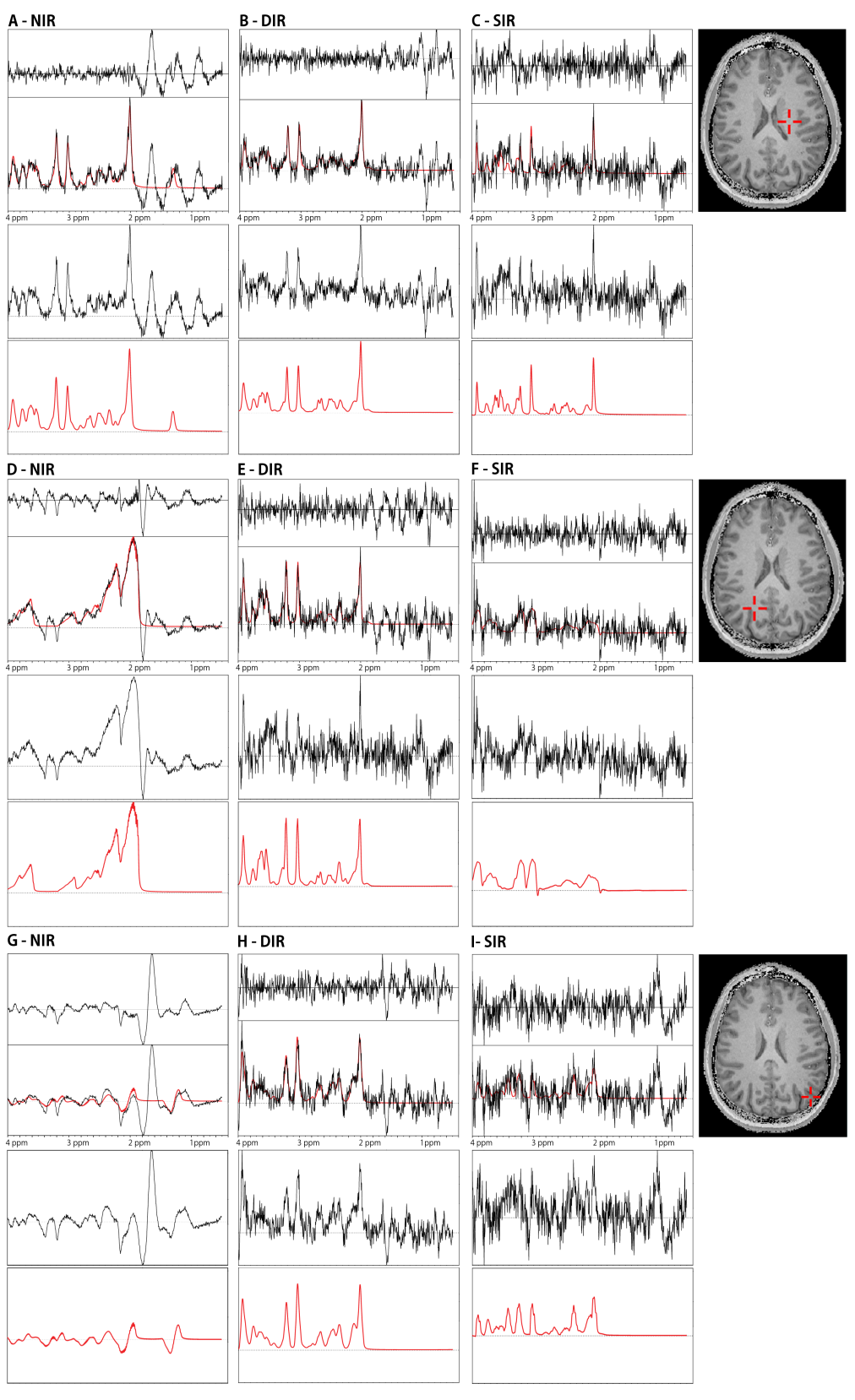


Figure for Review 1: The spectra presented figure 5 with a separate display of measured spectra (black) and LCModel fits (red). A-C: Spectra of a central white matter voxel for NIR, DIR, and SIR; D-F: spectra from the position of a lipid-fold-in artefact; G-I: spectra of a GM voxel in proximity to the cranium in the occipital cortex. Bottom plots: Measured spectra in black and LCModel fits in red. Top plots: Residuum of non-fitted signal. The spectral range of 0-4 ppm was processed.
