# Supplementary figures and images for: Lipid suppression via double inversion recovery with symmetric frequency sweep for robust 2D‐GRAPPA‐accelerated MRSI of the brain at 7 T
Source: NMR Biomed. 2015 Sep 15;28(11):1413–25. doi: 10.1002/nbm.3386 (PMC4973691; doi:10.1002/nbm.3386)

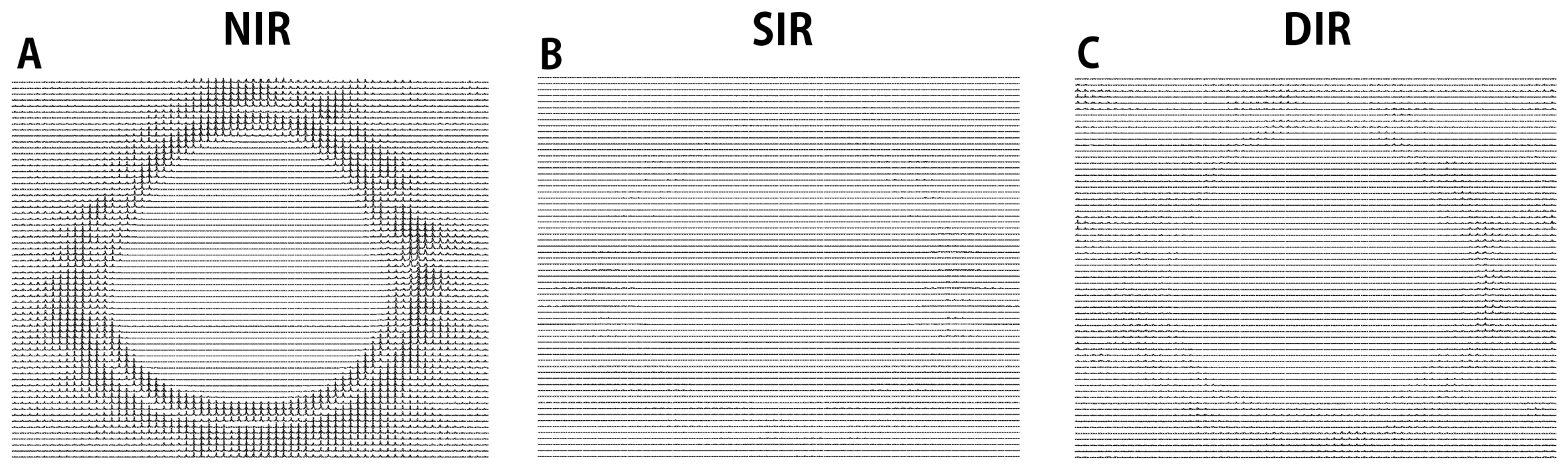

Supplement: Supplementary file 2 — Supporting info item [file NBM-28-1413-s002.tif]
